# Stable Isotope Labelling Reveals Water and Carbon Fluxes in Temperate Tree Saplings Before Budbreak

**DOI:** 10.1111/pce.15173

**Published:** 2024-10-01

**Authors:** Manuel G. Walde, Marco M. Lehmann, Arthur Gessler, Yann Vitasse, Haoyu Diao

**Affiliations:** ^1^ Ecosystem Ecology, Forest Dynamics, Swiss Federal Institute for Forest Snow and Landscape Research WSL Birmensdorf Switzerland; ^2^ Institute of Terrestrial Ecosystems ETH Zurich (Swiss Federal Institute of Technology) Zurich Switzerland; ^3^ Oeschger Centre for Climate Change Research University of Bern Bern Switzerland

**Keywords:** chilling, dormancy, forcing, stable isotope labelling, tree phenology, water allocation

## Abstract

Despite considerable experimental effort, the physiological mechanisms governing temperate tree species' water and carbon dynamics before the onset of the growing period remain poorly understood. We applied ^2^H‐enriched water during winter dormancy to the soil of four potted European tree species. After 8 weeks of chilling, hydrogen isotopes in stem, twig and bud water were measured six times during 2 consecutive weeks of forcing conditions (Experiment 1). Additionally, we pulse‐labelled above‐ground plant tissues using ^2^H‐enriched water vapour and ^13^C‐enriched CO_2_ 7 days after exposure to forcing conditions to trace atmospheric water and carbon uptake (Experiment 2). Experiment 1 revealed soil water incorporation into the above‐ground organs of all species during the chilling phase and significant species‐specific differences in water allocation during the forcing conditions, which we attributed to differences in structural traits. Experiment 2 illustrated water vapour incorporation into all above‐ground tissue of all species. However, the incorporation of carbon was found for evergreen saplings only. Our results suggest that temperate trees take up and reallocate soil water and absorb atmospheric water to maintain sufficient above‐ground tissue hydration during winter. Therefore, our findings provide new insights into the water allocation dynamics of temperate trees during early spring.

## Summary statement

Our stable isotope labelling study demonstrates that tree saplings take up soil and atmospheric water to hydrate above‐ground tissues before budburst. However, no carbon uptake was found for the investigated deciduous species during this period.

## 1 Introduction

The start of the growing period of temperate trees is mainly regulated by cool temperatures during winter necessary for dormancy release (chilling), warmer temperatures in spring (forcing) and day length (photoperiod), all of which interact with each other in intricate ways (reviewed in Delpierre et al. 2016). These factors have been the subject of numerous experiments, revealing substantial differences between species (e.g., Flynn and Wolkovich 2018; Laube et al. 2014; Walde et al. 2022). Once all the phenological requirements are met, water supply is critical to sustain cell expansion and division in bud meristems and enable budburst and leaf‐out regardless of species. However, it is largely unknown whether the water supply for cell expansion during bud swelling and budburst comes from adjacent tissues or the roots. It is known that temperate trees decrease their metabolic function and water transport by shutting down water transport channels at the onset of endodormancy to protect sieve tubes (phloem conduits) from frost damage and vessels (xylem conduits) from embolism triggered by freezing (Cavender‐Bares 2005; Cavender‐Bares et al. 2005; Cochard and Tyree 1990). Vascular water transport into buds is therefore assumed to be absent during the endodormancy either due to obstruction of water flow or immature xylem and appears to be restored only shortly before budburst (Goodwin 1967; Walde et al. 2024; Xie, Forney, and Bondada 2018).

In contrast, non‐vascular water transport, which refers to water movement through the plant's cells rather than through specialized transport tissues, could maintain bud hydration during dormancy by reallocating plant internal water from neighbouring tissues (Savage and Chuine 2021). The hydration of buds during winter dormancy appears particularly important since buds lose water during winter through the cuticle (i.e., nonstomatal transpiration) when a large humidity gradient occurs between air and inner bud tissue (Wiegand 1906). While existing studies recognize the importance of water supply to buds during winter, the transition point when trees switch from mainly non‐vascular to vascular transport processes to support meristematic cell divisions and elongation remains unclear (e.g., Götz and Chmielewski 2023; Vimont et al. 2021; Yooyongwech et al. 2008). Xie, Forney, and Bondada (2018) discovered that non‐vascular water transport initiates bud swelling of *Vitis vinifera* before the vascular connection between the bud and cane is established. Although non‐vascular water transport may contribute to cell expansion inside the bud, a functional vascular system would be essential to meet the water demand of temperate tree leaves later on (Lavrič et al. 2017; Zweifel et al. 2006). Laube et al. (2014) suggested that water vapour absorption (i.e., reversed transpiration) or uptake of water droplets through the plant surface could contribute to tissue rehydration during spring phenology. Mayr et al. (2014), for instance, showed that the evergreen coniferous species *Picea abies* can restore the water content of the embolized branch xylem by foliar water uptake, although this process seems to be unlikely to trigger leaf‐out of deciduous species (Zohner et al. 2020). However, investigations that consider dormancy release and water transport simultaneously are rare (but see Fouché et al. 2023). Therefore, tracking water transport processes during dormancy release can improve our understanding of spring phenological drivers (Hänninen et al. 2019), which have the potential to refine existing process‐based spring phenological models (e.g., Zhang et al. 2023).

Carbon fixation is another indispensable factor for plant development besides water and nutrient uptake. Trees fix carbon primarily through leaf photosynthesis, but bark photosynthesis is prevalent and represents an alternative way of fixing carbon (see Salomón et al. 2024), especially during unfavourable climatic conditions (reviewed in Ávila, Herrera, and Tezara 2014). Bark photosynthesis might thus be happening during the winter dormancy of temperate tree species (Wittmann and Pfanz 2007).

Nevertheless, temperate tree species differ substantially in their physiological processes regarding water and carbon supplies during winter dormancy. Among the most evident examples are evergreen species that require more water to hydrate their leaves during winter than deciduous species. Further, morphological differences, such as wood anatomy during winter dormancy, could also affect the hydration of deciduous tree tissues. Essiamah and Eschrich (1986) suggested that the onset of root water uptake and vascular transport processes for diffuse‐porous species accompanies bud swelling during spring. In contrast, ring‐porous species rebuild new‐conducting vessels before leaf phenology during spring (e.g., Sass‐Klaassen, Sabajo, and den Ouden 2011) and the vascular transport systems only become functional during budburst. Although plant internal water allocation in trees at the initiation of spring is somewhat understood, it is mainly unknown if bark photosynthesis of temperate tree species could fix a traceable amount of CO_2_ from the atmosphere during winter or if tree buds contribute to carbon assimilation.

Here, we aimed at tracking water and carbon (re)‐allocation processes focusing on stem, twig and bud tissues towards the end of winter dormancy, that is, shortly before budburst. For this purpose, we injected highly enriched ^2^H‐water into the soil of four potted tree species with contrasting physiological traits during winter dormancy. Using this isotopic labelling method, we could track potential water transport from soil to above‐ground tissues before budburst and quantify changes in tissue water content after exposure to forcing conditions (Experiment 1). In addition, we also quantified atmospheric water absorption and carbon fixation into above‐ground tissues by applying water vapour enriched in ^2^H and carbon dioxide (CO_2_) enriched in ^13^C before budburst to two deciduous and one evergreen tree species (Experiment 2).

Our methodology enabled us to trace root water uptake and plant internal water allocation during experimental chilling and forcing conditions. Further, we determined the water and carbon allocation of different above‐ground tissues (i.e., stem, buds and needles) after 1 week of forcing conditions during spring. Our two experiments addressed the following three hypotheses.

1. Cold temperatures largely restrain root water uptake and prevent below‐ground to above‐ground water allocation processes for all tree species. Therefore, we expect water flow restoration from soil to buds during spring to largely depend on species‐specific tree physiological characteristics during dormancy release.
2. Trees lose water during winter through the cuticle and lenticels, dependent on atmospheric evaporative demand. We hypothesize that this process could be reversed under low atmospheric demand so that trees could absorb atmospheric water before budburst.
3. Bark photosynthesis is typical for a set of tree species. Therefore, we hypothesize carbon assimilation to occur in traceable quantities for deciduous broad‐leaf species before spring budburst.

## 2 Materials and Methods

### 2.1 Study Species

We selected four tree species native to central Europe with contrasting resource‐use strategies: *Fagus sylvatica* L., a shade‐tolerant deciduous broad‐leaf species prevalent in a humid temperate climate; *Quercus petraea* Liebl. and *Sorbus torminalis* L., both deciduous broad‐leaf species that predominantly grow in dry‐warm and sparse forests due to their markedly higher drought tolerance and higher light requirement compared to *F. sylvatica* and *Pinus sylvestris* L., a light‐demanding evergreen coniferous species with relatively low nutrient and water demands. All species differ in their wood anatomical traits: *F. sylvatica* is a diffuse‐porous to semi‐ring‐porous species, *Q. petraea* is a ring‐porous species, *S. torminalis* is a diffuse‐to slightly semi‐ring‐porous and *P. sylvestris* is a coniferous species with tracheids (Schoch et al. 2004). Depending on the species, the experiment was conducted with potted saplings from Swiss nurseries aged 2–5 years and 50–87 cm tall (Supporting Information S1: Table S1).

### 2.2 Experiment 1: Below‐Ground ^2^H_2_O‐labelling During Winter Dormancy

**Figure 1:**
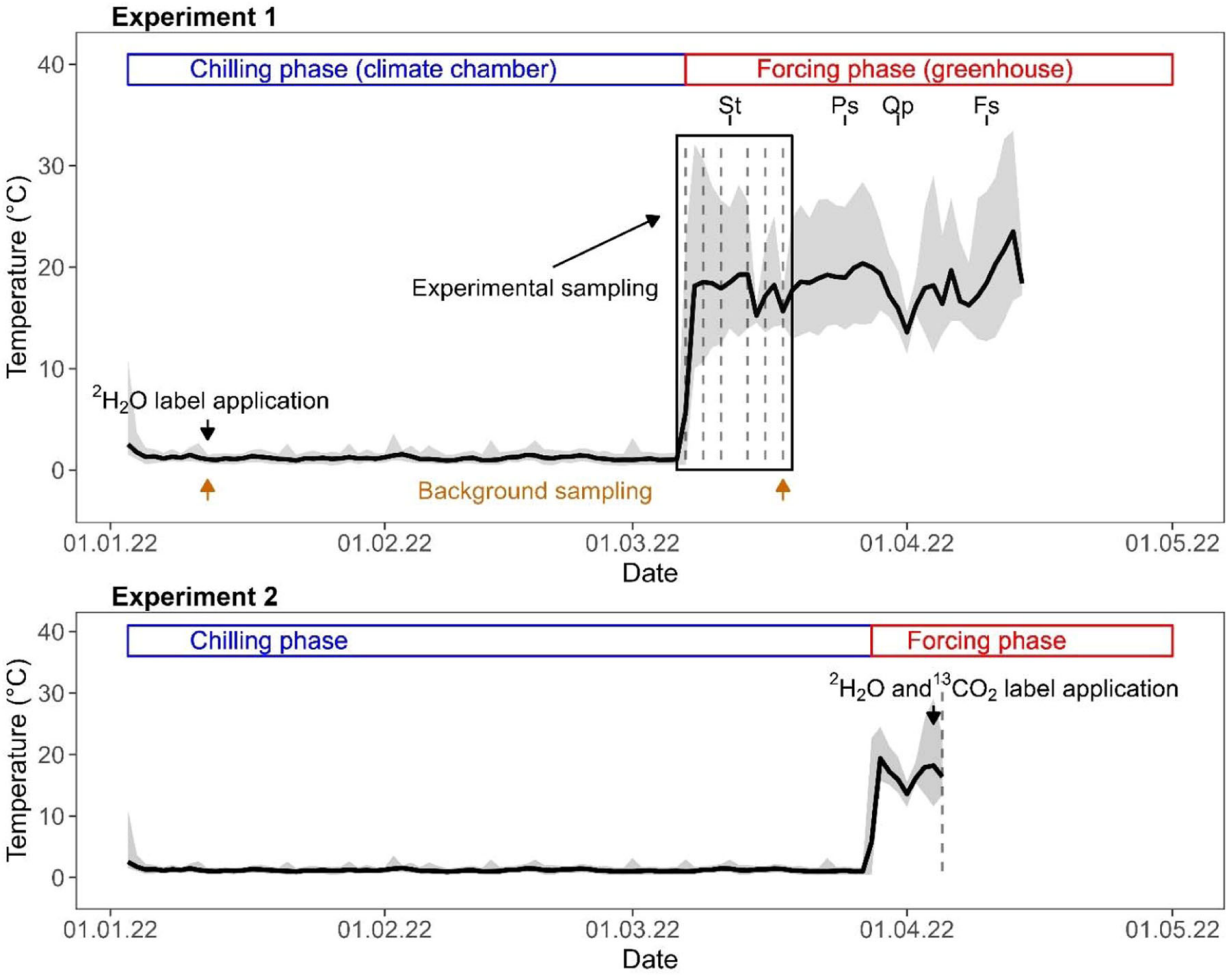
Temperature exposure of the potted tree saplings during the chilling (climate chamber) and forcing phase (greenhouse). The grey areas represent the area between the daily minimum and maximum temperatures, and the thick black line corresponds to the daily mean temperature. Experiment 1: The sampling campaigns are illustrated with dashed lines and were executed at regular intervals within 2 weeks after initiation of the forcing phase. The black arrow indicates the date of the ^2^H_2_O label application, whereas the orange arrows indicate the dates of background sampling. The timing of budburst of *Sorbus torminalis*, *Pinus sylvestris*, *Quercus petraea* and *Fagus sylvatica* is illustrated as St, Ps, Qp and Fs, respectively, and was determined for Experiment 1 only.

All saplings were kept outside at the experimental tree nursery of the Swiss Federal Institute for Forest, Snow and Landscape Research (WSL, 47°21′38″ N, 8°27′16″ E; 550 m asl, average annual temperature at the weather station Zürich/Kloten 9.8°C from 1987 to 2016) since February 2021 and watered daily during summer 2021. In the first week of 2022, all potted saplings were moved from ambient outdoor conditions to a climate chamber (Bitzer 6HE‐35Y, Kälte 3000 AG, Landquart, Switzerland) with constant cold temperature (1.2 ± 0.2°C) to fully satisfy the chilling requirement for dormancy release (chilling phase, Figure 1). This chilling phase lasted from 3 January to 7 March 2022. After 1 week of chilling inside the climate chamber, that is, on 10 January, 40 mL of ^2^H‐labelled water (*δ* ^2^H = 2037 ± 8‰) was injected into each of the pots of 36 *S. torminalis* and *F. sylvatica* saplings, whereas 36 saplings of *Q. petraea* and *P. sylvestris* got 30 and 20 mL for each pot, respectively, due to their smaller pot sizes and lower water retention capacities. The injection of the ^2^H_2_O label into the soil of the tree sapling enriched the isotopic composition of the soil ranging from 273 ± 213 to 554 ± 208‰, depending on species (see Supporting Information S1: Table S2) From then onwards, labelled tree saplings were not watered until the destructive samplings to quantify stable water isotopes of the tissue water. The ^2^H‐label uptake and reallocation were analyzed for all species individually due to species‐specific differences in soil water *δ* ^2^H.

On 7 March, all 36 ^2^H_2_O‐labelled saplings and nine non‐labelled saplings of all species were transferred from the cold climate chamber into a greenhouse and exposed to warm air temperatures. Inside the greenhouse, the tree saplings were exposed to direct sunlight without shading (ambient irradiation from 7 to 18 March was about 141 and 560 W/m^2^ for daily mean and maximum, respectively). From 7 to 18 March, six individuals from labelled pots were destructively sampled for each species every second or third day (i.e., 0, 2, 4, 7, 9 and 11 days after exposure to forcing conditions) to quantify the ^2^H_2_O‐label taken up from the soil and the tissue water content. *P. sylvestris* was, in contrast to the other three investigated species, only sampled on 7, 9 and 11 March (i.e., 0, 2 and 4 days after exposure to forcing conditions) as the pots of this species dehydrated faster after exposure to forcing conditions due to the presence of needles that transpired significantly. We wanted to avoid drought stress impacting our measurements and stopped Experiment 1 for the remaining *P. sylvestris* saplings after 4 days.

Air temperature during the chilling and forcing phase was recorded every 30 min by HOBO temperature loggers (HOBO MX2203, Onset Computer Corporation, Bourne, MA, USA). The average temperature during the chilling phase (i.e., from 3 January to 7 March) was 1.2 ± 0.2°C (mean ± SD). We chose this temperature protocol to satisfy the chilling requirement while minimizing accumulation of forcing temperatures (Chuine 2000; Walde et al. 2022). The daily average temperature during the forcing phase (from 7 to 18 March) was 17.8°C (minimum 13.0°C, maximum 25.4°C), which roughly corresponds to the ambient air temperature at unusual warm April days or the long‐term average temperature recorded by the institute at the beginning of June. Therefore, the temperature protocol is exerting strong, yet realistic, forcing conditions on the tree saplings. Budburst of *S. torminalis* occurred during the sampling period less than 1 week after exposure to forcing conditions (11–13 March). In contrast, budburst of *P. sylvestris*, *Q. petraea and F. sylvatica* occurred about 20 days (24–29 March), 23 days (29–31 March) and 33 days (7–12 April) after exposure to forcing conditions, respectively (Figure 1).

At each sampling date, stem, twig and bud tissues were destructively sampled on six individuals for each species. Stem samples were cut off at about 3–5 cm above the soil, while twigs and buds were sampled from the uppermost 10 cm of the saplings' canopy (Supporting Information S1: Figure S1). We sampled at least five twigs and buds from at least three branches for each individual. The twig samples consisted of 1 cm twig below the bud, while the buds were sampled about 2 mm above the twig‐bud crossing. After abscission, the samples were stored at −20°C until water extraction for isotope analyses. We included 1‐year‐old needles into the twig samples of *P. sylvestris* to separate newly forming photosynthetic tissues (i.e., inside the buds) from existing, functional tissue to reflect natural circumstances.

Three non‐labelled individuals for each species were sampled on 10 January (i.e., after 1 week of chilling) and another set of three individuals on 18 March (i.e., after 11 days at forcing conditions) to calibrate the background relationship of the hydrogen (*δ* ^2^H) and oxygen isotope compositions (*δ* ^18^O) for all tissues (see Section 2.5). In addition, six non‐labelled individuals for each species were exposed to the same chilling and kept at forcing conditions until the spring phenology of all trees was completed. Spring phenological development of the respective species was assessed using a five‐stage categorical scale three times a week to determine the budburst date (see Vitasse 2013).

### 2.3 Experiment 2: Above‐Ground ^2^H_2_O‐ and ^13^CO_2_‐labelling

For Experiment 2, six saplings of *Q. petraea, F. sylvatica* and *P. sylvestris* were kept in the cold climate chamber until 27 March and exposed to forcing conditions on 28 March in the same greenhouse already used for Experiment 1 (Figure 1). *S. torminalis* was excluded from Experiment 2 as this species started to budburst within 4 days in forcing conditions in Experiment 1, and we aimed to investigate hydraulic processes acting before budburst. After 1 week of forcing conditions (mean temperature 16.9°C, daily minimum 13.8°C, daily maximum 22.1°C), in the evening of 4 April, the pots of the saplings were wrapped with transparent plastic bags and fixed with rubber bands to prevent from any water vapour exchange between the soil and the air. Then, the saplings were moved from the greenhouse into a tent installed inside a climate chamber (Conviron PGR15, Controlled Environments Limited, Manitoba, Canada) and kept there for the night.

On 5 April, the atmospheric labelling was conducted from 9:00 AM to 3:30 PM. No visible sign of spring phenology was observed for any of the study species during Experiment 2. The above‐ground dual isotope pulse‐labelling was applied by pumping water vapour enriched in the heavier hydrogen isotope (^2^H_2_O) and carbon dioxide enriched in the heavier carbon isotope (^13^CO_2_) into the tent (Studer et al. 2015; Wang et al. 2021). The air temperature (*T*) inside the climate chamber was 19.7 ± 1.0°C, and the plants were exposed to a photon flux density of 489 ± 19 µmol/m^2^/s (Supporting Information S1: Figure S2A,B. Relative air humidity (RH) was measured inside the chamber and calculated inside the tent (see Supporting Information). The RH inside the climate chamber was 63 ± 14% during the atmospheric labelling, while the RH inside the tent was just above saturation at 102 ± 16% on average (Supporting Information S1: Figure S2C).

CO_2_ concentration inside the tent was 564 ± 54 ppm throughout the above‐ground labelling (Supporting Information S1: Figure S2D). The stable isotopic composition of the air in the tent was continuously registered using a CO_2_ isotope analyzer (Delta Ray, Thermo Fisher Scientific Inc., Bremen, Germany) and a H_2_O isotope analyzer (L2120‐i, Picarro Inc., Santa Clara, CA, USA) during the labelling. Compared with the non‐labelling periods, both *δ* ^2^H and *δ* ^13^C increased strongly during the above‐ground labelling with 491 ± 247‰ for *δ* ^2^H and 6992 ± 4845‰ for *δ* ^13^C (Supporting Information S1: Figure S2E,F).

After the atmospheric labelling ended, plants were moved out of the climate chamber and returned to the forcing condition inside the greenhouse previously used. After 1 h, stem, twig and bud tissues were harvested as in Experiment 1. Before the harvest, 2 g from the uppermost 5 cm of soil was taken from three pots of each species to control for any contamination of ^2^H_2_O penetrating the plastic bags and getting into the soil, which was found to be negligible (Supporting Information S1: Figure S3).

### 2.4 Water Extraction and Stable Isotope Analysis

A self‐made cryogenic vacuum distillation (CVD) based on the model described by West, Patrickson, and Ehleringer (2006) was used to extract water enclosed in the samples for water isotope analyses. The main components of the CVD system utilized are a water bath maintained at 80°C with samples submerged in and u‐tubes submerged in liquid nitrogen for collecting extracted water (see Diao et al. 2022; Orlowski et al. 2013). The process was accelerated with a vacuum pump (BS2212, Brook Crompton Ltd, Doncaster, UK) that kept the pressure below 0.05 mbar during the extraction. The extraction was carried out for 2 h, and after the extraction, the whole system was flushed with dry nitrogen gas before the u‐tubes were detached. Then, the u‐tubes were sealed with rubber plugs, and the extracts were thawed at room temperature before transferring them into glass vials (Infochroma AG, Goldau, Switzerland) using a pipette. Sample weights were determined before and after the extraction to determine the water content of the samples. On average, 0.91 ± 0.29, 0.32 ± 0.34 and 0.24 ± 0.21 mL of plant tissue waters was extracted from stems, twigs and buds, respectively.

Stable water isotopes of the extracts, *δ* ^2^H and *δ* ^18^O, were measured with a high‐temperature conversion elemental analyzer coupled to a Delta^Plus^ XP isotope ratio mass spectrometer (TC/EA‐IRMS; Finnigan MAT, Bremen, Germany) with a precision of 1‰ for *δ* ^2^H and 0.2‰ for *δ* ^18^O.

After water extraction, the dry tissues of the control trees and the trees used in Experiment 2 were ground to a fine powder using an ultracentrifugal mill (ZM1000; Retsch, Haan, Germany). Then, 1 ± 0.1 mg of dry bulk sample was weighted in tin capsules (3.3 × 5 mm, Säntis Analytical AG, Teufen, Switzerland) using a high precision balance (MT5; Mettler‐Toledo, Greifensee, Switzerland), and the samples were analyzed for *δ* ^13^C using an EA‐IRMS (Thermo EA 1100 Delta^plus^ XL; 0.1‰ precision).

### 2.5 Background Correction of *δ* ^2^H

A stable linear relationship between *δ* ^2^H and *δ* ^18^O in tissue water is generally found under natural conditions and can be expressed in the form of:

(1) $$\delta^{2}H={b+a\times \delta }^{18}O.$$

First, a linear relationship between *δ* ^2^H and *δ* ^18^O was determined for six non‐labelled saplings after 7 days of the chilling phase and at the last sampling date of the forcing phase (*R* ^2^ = 0.99, see Supporting Information S1: Figure S4). Then, this relationship was used in the next step to predict the *δ* ^2^H values of each labelled sapling that were not affected by the labelling (*δ* ^2^H_background_) at each sampling date using the measured *δ* ^18^O values (*δ* ^18^O_sample_) as the *δ* ^18^O in the water used for labelling is isotopically very close to the water used for irrigation in the experiments

(2) $$\delta^{2}H_{\mathrm{background}}={b+a\times \delta }^{18}O_{\mathrm{sample}}.$$

Then, the predicted *δ* ^2^H_background_ was subtracted from the measured hydrogen isotope composition of the tissues sampled after the labelling (*δ* ^2^H_sample_) to determine ^2^H_2_O‐label excess (Δ^2^H, Equation 3), which represent the focus of the two experiments of our study

(3) $$\Delta^{2}H=\delta^{2}H_{\mathrm{sample}}-\delta^{2}H_{\mathrm{background}}.$$

In addition, we approximated the fraction of incorporated label into plant tissues to have a better representation of the actual amount of labelled water in each tissue (see Equations S4–S6 of the Supporting Information).

### 2.6 Data Analysis and Statistics

All analyses and plots were performed in R v.4.1.1 (R Core Team 2023). *δ* ^2^H was modelled by linear regression with *δ* ^18^O (continuous variable), species (categorial variable), tissue (categorial variable) and every possible two‐way and three‐way interaction as fixed effects using the *stats* package of base R. Species‐ and tissue‐specific regression lines and corresponding confidence intervals were predicted using *ggeffects* (v1.2.3). The correlation between tissue water content, tissue ^2^H_2_O‐label excess (Δ^2^H) and exposure to forcing conditions were evaluated species‐specific and tissue‐specific using Pearson's correlation coefficient in Experiment 1. Species‐specific differences label excess differences between tissues in Experiments 1 and 2 were evaluated using two‐sided Wilcoxon signed rank tests. Treatment‐specific differences between tissue *δ* ^13^C in Experiment 2 were evaluated using one‐sided Wilcoxon signed ranks tests in the atmospheric labelling experiment. Correlations and non‐parametric Wilcoxon signed rank tests were executed using the pipe‐friendly R package *rstatix* (v0.7.2). All figures were generated using *ggplot2* (v3.4.2).

## 3 Results

### 3.1 Experiment 1: Root Water Uptake and Water Allocation

**Figure 2:**
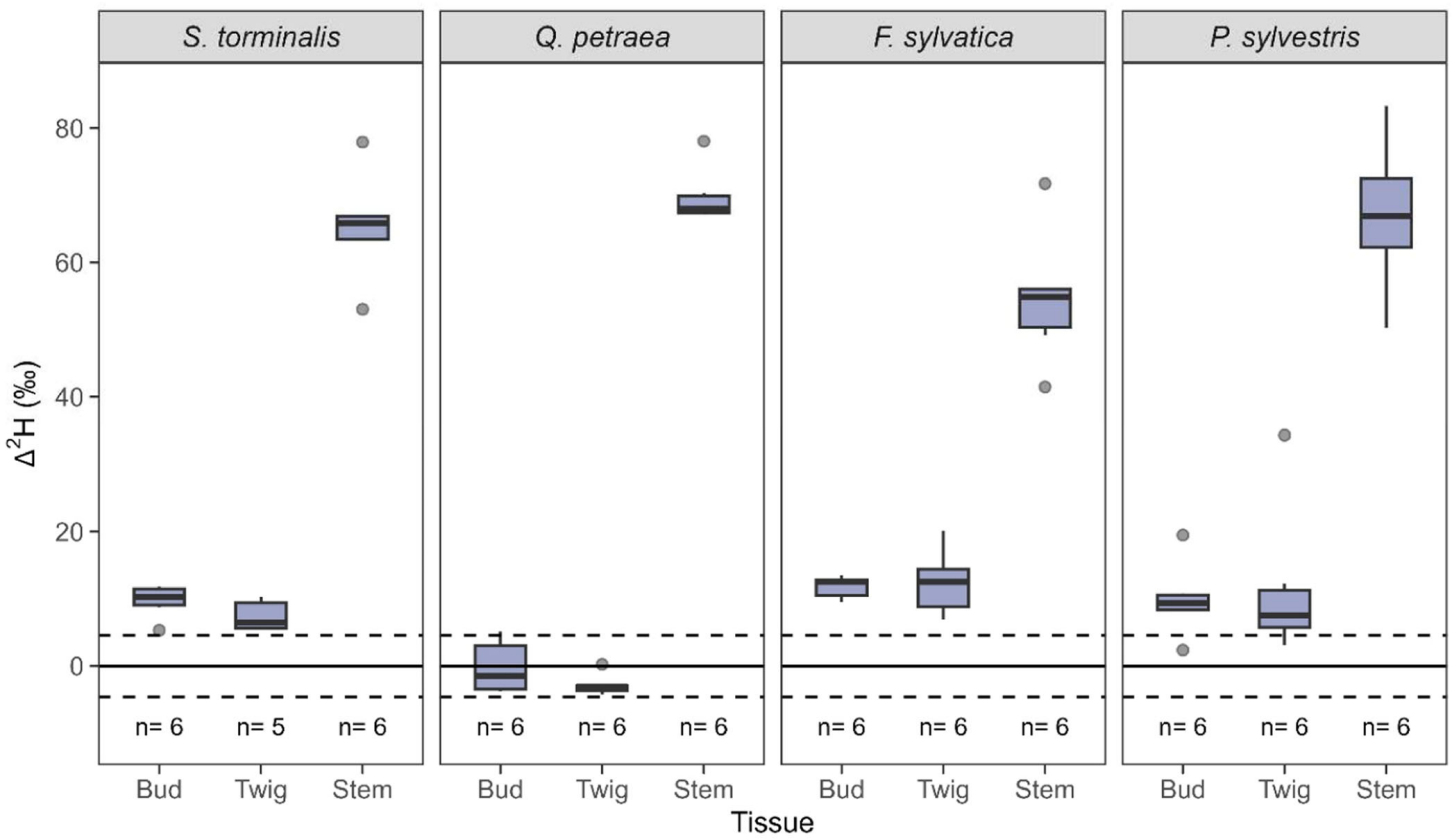
^2^H_2_O‐label excess (Δ^2^H) at the initiation of the forcing phase of Experiment 1 in different above‐ground tissues after exposure to soil water enriched in ^2^H. The boxes represent the first quartile, median and third quartile, while the whiskers indicate 1.5 times the interquartile range in both directions and are aggregated in species. The dots represent measurements outside the whisker range. The solid and dashed lines represent the background's mean and mean standard error of 4.6‰, respectively, *n* represents the number of replicates. [Color figure can be viewed at wileyonlinelibrary.com]

Water contained in the stem tissue of all investigated individuals was enriched in ^2^H compared with the background at the initiation of the forcing phase (Figure 2). Thus, some ^2^H_2_O‐label was already incorporated into the lower stem at the end of the chilling phase while the tree saplings were exposed to ∼1°C. However, species differed in water allocation from stem to twigs and buds during the chilling phase. All twig and bud Δ^2^H values of *F. sylvatica* and *S. torminalis* saplings were higher than the standard error of the background calibration of +4.6‰ at the end of the chilling phase (Figure 2), which indicates some water transport from the lower stem to above‐ground organs at temperatures close to freezing for these species. One out of six *P. sylvestris* saplings had twig and bud Δ^2^H values lower than +4.6‰, whereas no labelled water was detected in these tissues for *Q. petraea*. Thus, except *Q. petraea* all species took up approximately 10%–15% of labelled water from the soil into the stem tissue and 1%–3% into twig and bud tissues during the chilling phase of Experiment 1, whereas a higher soil water uptake into stem and no soil water uptake into twigs and buds was observed for *Q. petraea* during this period (Supporting Information S1: Table S2).

Bud water content of *S. torminalis* increased (*r* = 0.76, *p* < 0.001) and stem water content of *P. sylvestris* decreased (*r* = −0.79, *p* < 0.001) with increasing exposure to forcing conditions (Supporting Information S1: Figure S5). Further, we found weak and marginally significant evidence for increasing stem water content of *Q. petraea* (*r* = 0.33, *p* = 0.052) and increasing twig water content of *P. sylvestris* (*r* = 0.45, *p* = 0.063) with increasing exposure to forcing conditions. The water content of all other tissues was unaffected across species with increasing exposure to forcing conditions (Supporting Information S1: Figure S5).

**Figure 3:**
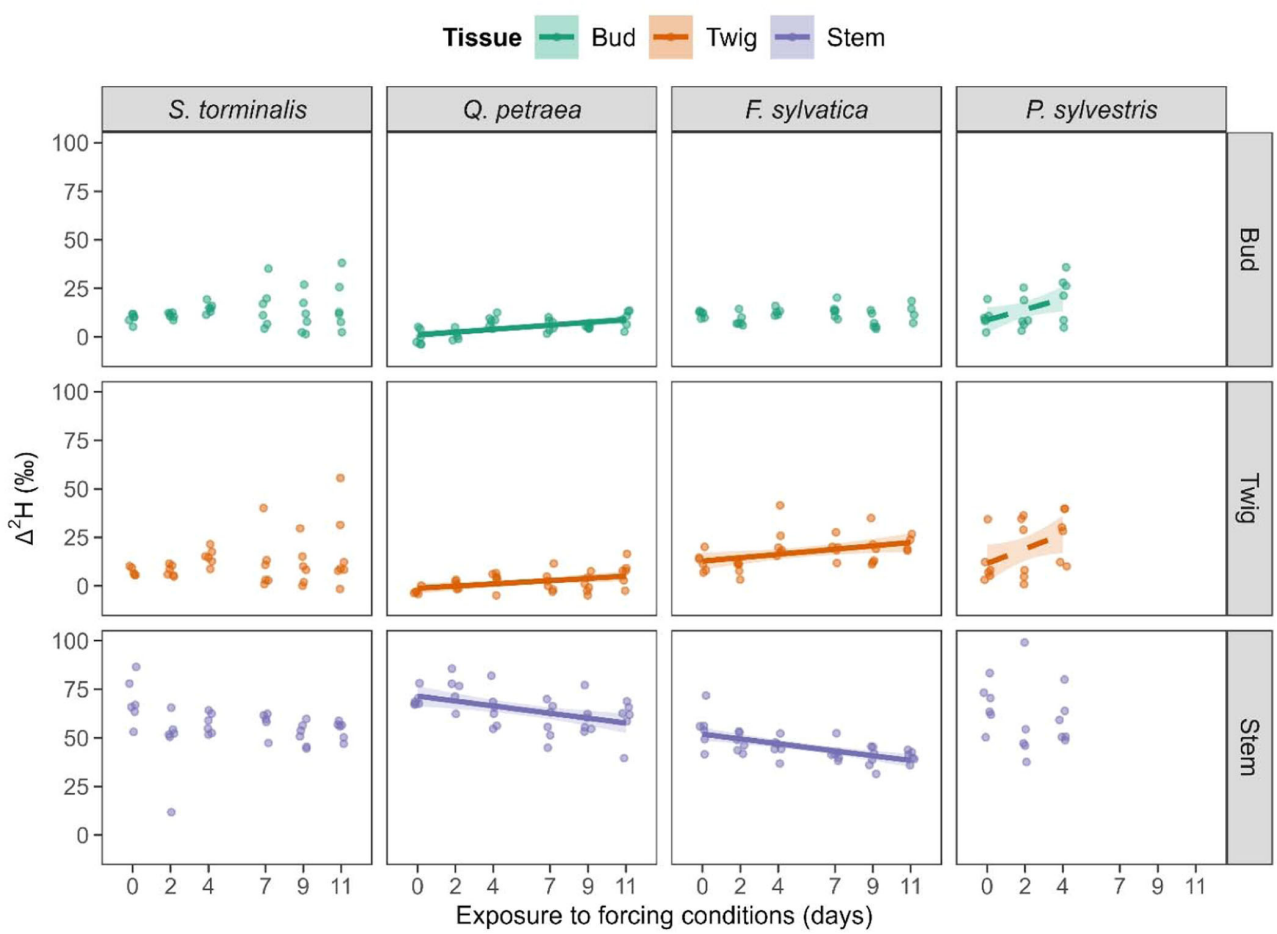
Change in Δ^2^H with increasing forcing conditions in Experiment 1 for each studied species and tissue. Regression lines with corresponding 0.95 confidence intervals depict the direction of the correlations. Solid lines represent significant correlations (*p* ≤ 0.050), while dashed lines represent correlations at 0.050 < *p* ≤ 0.100. [Color figure can be viewed at wileyonlinelibrary.com]

All species showed a higher stem Δ^2^H than twigs and buds Δ^2^H throughout the forcing phase (Figure 3). Stem Δ^2^H of *Q. petraea* and *F. sylvatica* decreased (*r* = −0.49, *p* = 0.003 and *r* = −0.64, *p* < 0.001, respectively) while twig Δ^2^H of these two species increased with increasing forcing (*r* = 0.44, *p* = 0.008 and *r* = 0.41, *p* = 0.018, respectively). Bud Δ^2^H increased significantly in *Q. petraea* buds but not in the buds of any other investigated species (*r* = 0.61, *p* < 0.001). This trend at a time when the buds are not yet swollen indicates a dynamic of upward water flow from roots to buds without leaf transpiration.

A distinct but marginally significant trend toward increasing bud and twig Δ^2^H with increasing exposure to forcing conditions was found for *P. sylvestris* (*r* = 0.47, *p* = 0.051 and *r* = 0.44, *p* = 0.066, respectively), whereas *S. torminalis* did not show such a trend (Figure 3). *S. torminalis* showed a relatively low Δ^2^H diffusion of twig and bud water during the first 4 days after exposure to forcing conditions, while the diffusion of Δ^2^H of both tissues was much stronger afterwards (Figure 3). No correlation between stem and twig label excess was found for any species (Supporting Information S1: Figure S6A). However, a strong correlation between twig and bud label excess was found for all species but *F. sylvatica* (Supporting Information S1: Figure S6B), indicating that *S. torminalis*, *Q. petraea* and *P. sylvestris* are less restricted in the water flow from twig into buds towards the end of winter than *F. sylvatica*.

**Figure 4:**
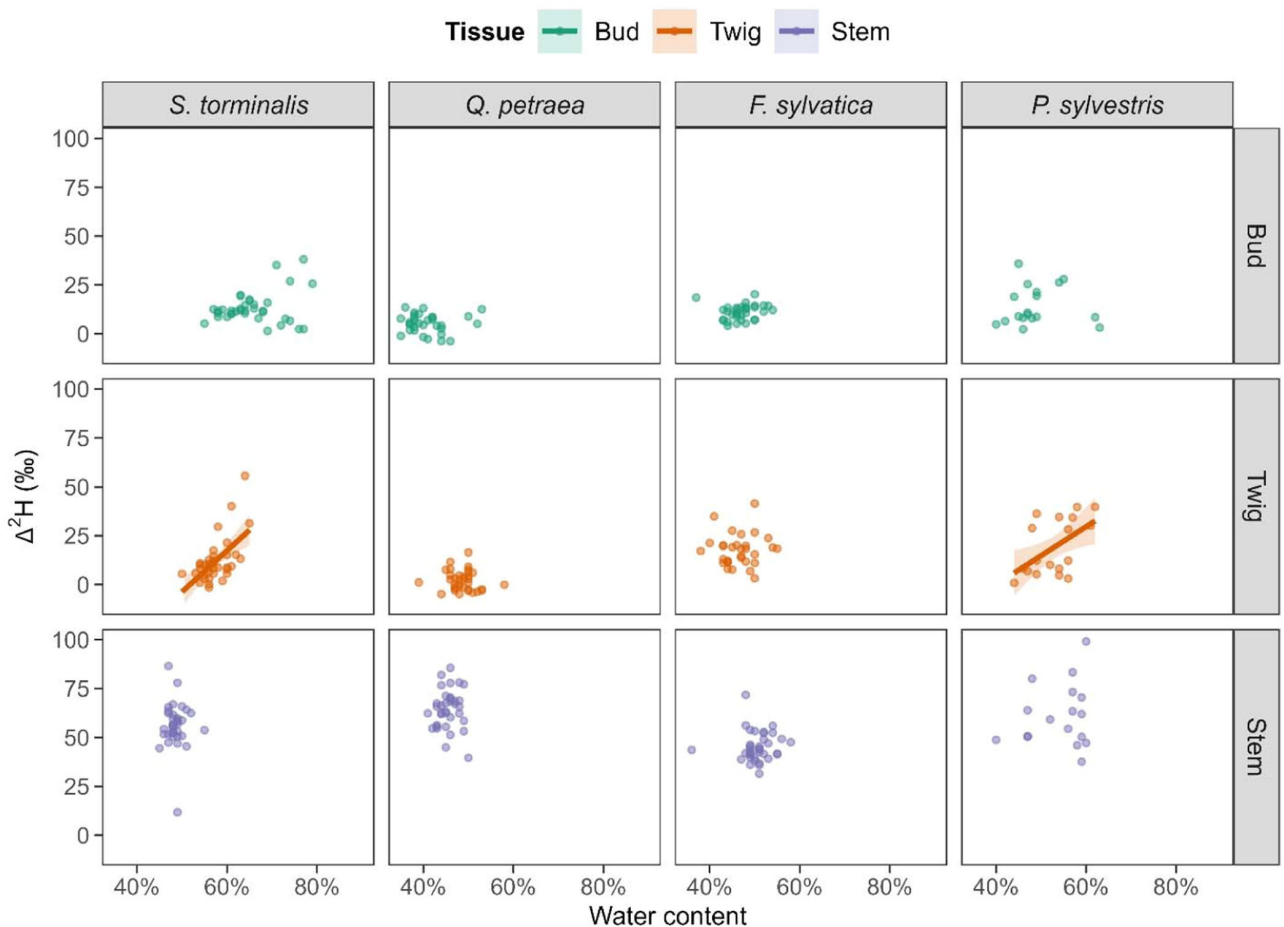
Change in Δ^2^H with increasing water content in Experiment 1 for each studied species. Regression lines with corresponding 0.95 confidence intervals depict the direction of the correlations. Solid lines represent significant correlations (*p* ≤ 0.050). [Color figure can be viewed at wileyonlinelibrary.com]

Bud and stem Δ^2^H did not correlate with water content for any species (Figure 4). However, twig Δ^2^H significantly increased with increasing water content in *S. torminalis* and *P. sylvestris* (*r* = 0.60, *p* < 0.001 and *r* = 0.54, *p* = 0.021, respectively). This finding provides evidence for increasing twig water content being driven by an upward water flow during the forcing phase in the early flushing deciduous *S. torminalis* and evergreen *P. sylvestris*.

### 3.2 Experiment 2: Atmospheric Water Absorption

**Figure 5:**
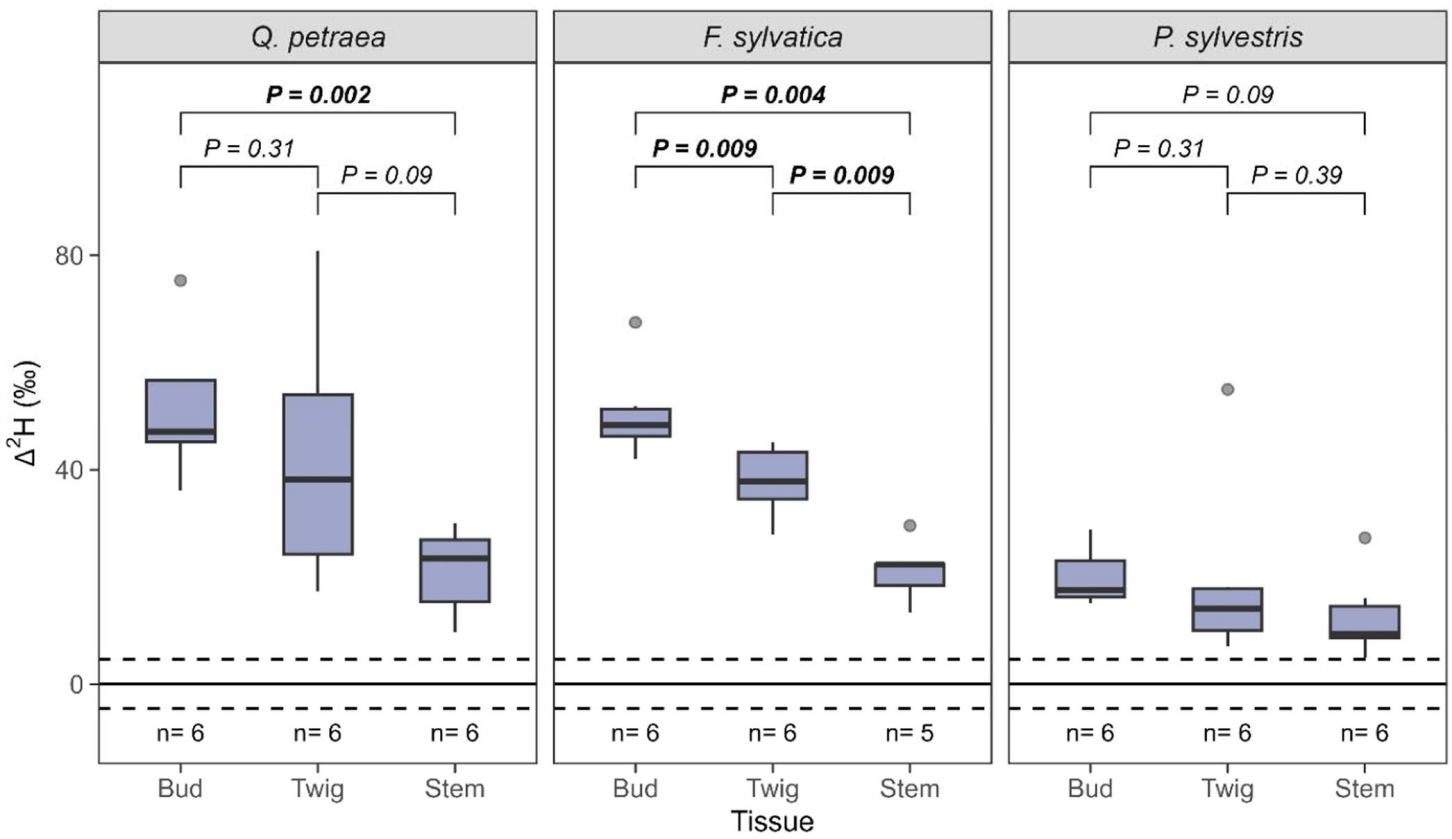
Δ^2^H in different above‐ground tissues in Experiment 2 after exposure to water vapour enriched in ^2^H aggregated in species. The boxes represent the first quartile, median and third quartile, while the whiskers indicate 1.5 times the interquartile range in both directions. The solid and dashed lines represent the background's mean and mean standard error of 4.6‰, respectively. *p* represents the *p* value of species‐specific two‐sided Wilcoxon signed rank tests between tissues with significant *p* values indicated in bold, *n* represents the number of replicates. [Color figure can be viewed at wileyonlinelibrary.com]

The atmospheric water vapour labelling results showed positive Δ^2^H in all species and tissues sampled (Figure 5), which reveals that all plants can absorb atmospheric water into their above‐ground tissues during winter dormancy under saturated humidity. Δ^2^H was significantly higher in buds than stem for *Q. petraea* and *F. sylvatica* saplings (*p* = 0.002 and *p* = 0.004, respectively), whereas no such difference between tissues was found for *P. sylvestris*. Further, all species showed a higher twig Δ^2^H than stem Δ^2^H and a lower twig Δ^2^H than bud Δ^2^H. However, this trend was only significant for *F. sylvatica* (*p* = 0.009 and *p* = 0.009, respectively). Thus, twig and bud water consisted of approximately 10%–13% and 7%–10% of labelled water uptaken from vapour for *Q. petraea* and *F. sylvatica*, respectively, whereas the water pool of those tissues consisted of about 4% of labelled water vapour for *P. sylvestris* (Supporting Information S1: Table S3).

### 3.3 Experiment 2: Carbon Assimilation

**Figure 6:**
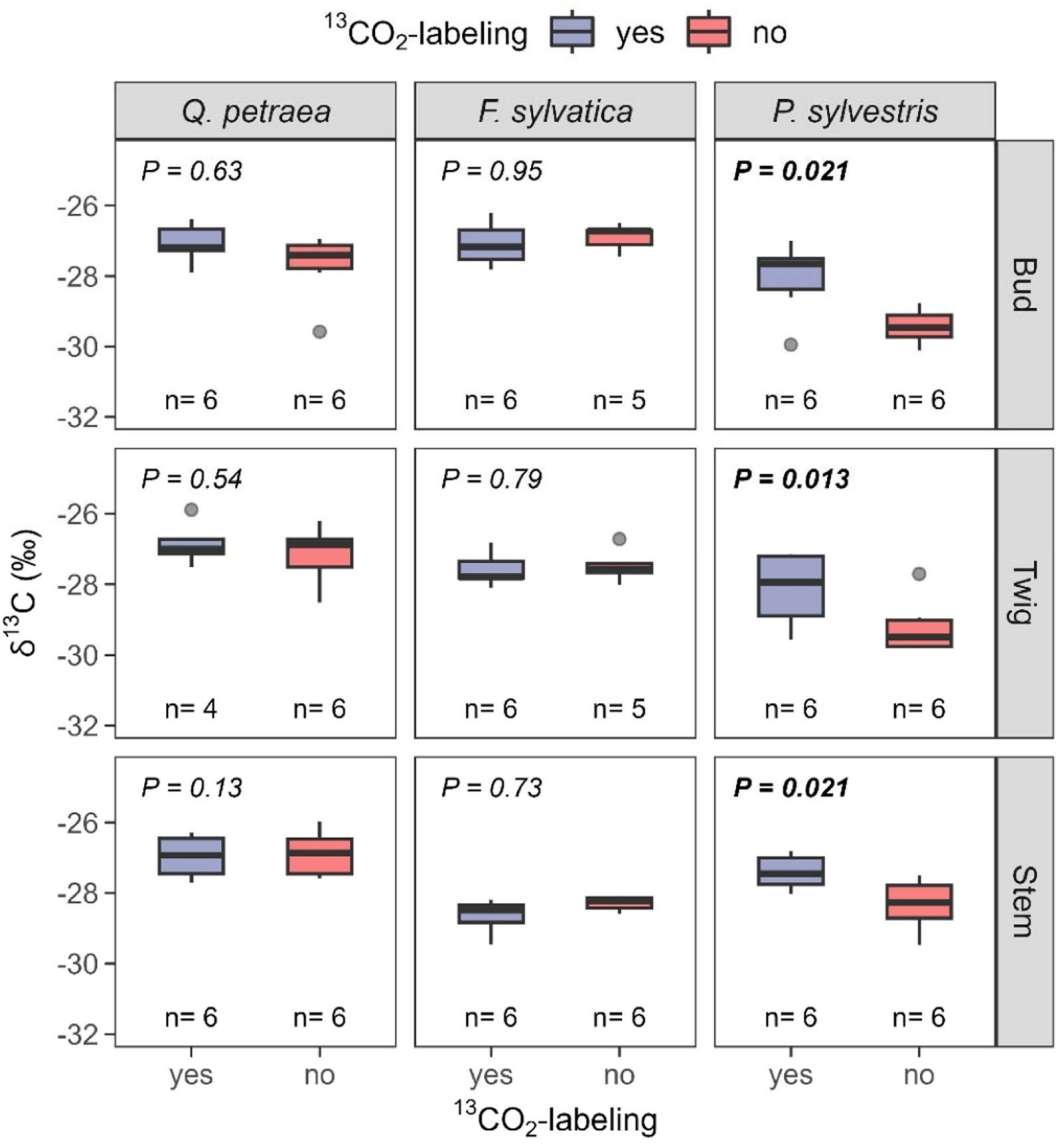
*δ* ^13^C in different above‐ground tissues in Experiment 2 after exposure to carbon dioxide enriched in ^13^C aggregated in species. The boxes represent the first quartile, median and third quartile, while the whiskers indicate 1.5 times the interquartile range in both directions. *p* represents the *p* value of tissue‐ and species‐specific one‐sided Wilcoxon signed rank tests between labelled and unlabelled individuals with significant *p* values indicated in bold, *n* represents the number of replicates. [Color figure can be viewed at wileyonlinelibrary.com]

None of the investigated tissues (i.e., buds, twigs and stem) of *Q. petraea* and *F. sylvatica* saplings were enriched in ^13^C. In contrast, the stem, twigs and buds of *P. sylvestris* were significantly enriched in ^13^C compared to the control trees after ^13^CO_2_ labelling (*p* = 0.021, *p* = 0.013 and *p* = 0.021, respectively; Figure 6). Thus, the evergreen *P. sylvestris* assimilated atmospheric CO_2_ 7 days after exposure to forcing conditions, likely with the needles from the previous years, whereas no evidence for substantial carbon assimilation was found for *Q. petraea* and *F. sylvatica*.

## 4 Discussion

The soil labelling of Experiment 1 revealed water uptake and reallocation to twigs and buds at temperatures slightly above freezing for all species (i.e., chilling phase). Interestingly, during the exposure to warm temperatures (i.e., forcing phase), stem and twig water content did not change in the deciduous broad‐leaf species *Q. petraea* and *F. sylvatica*. However, both species increased twig Δ^2^H with increasing exposure to forcing, suggesting an upward flow of ^2^H_2_O label from the stem to twigs xylem to maintain the water content. The early flushing deciduous *S. torminalis* and evergreen *P. sylvestris* showed a positive correlation between twig Δ^2^H and twig water content. This finding suggests that those species are allocating water from distant tissues through their vascular system to sustain bud swelling (*S. torminalis*) and transpiration of the needles (*P. sylvestris*) rather than solely counterbalancing the evaporation and transpiration water loss. The atmospheric labelling applied in Experiment 2 revealed that all investigated species could absorb atmospheric water into different above‐ground tissues (stem, twig and bud). However, unlike the evergreen species, the leafless deciduous species could not fix carbon in traceable concentrations before budburst.

### 4.1 Below‐Ground Water Uptake Keeps Above‐Ground Tree Tissues Hydrated During Winter

Contrary to our expectations, all species took up labelled water into their stem during the chilling phase. This finding indicates root water uptake and water flow from roots to stem at temperatures just above freezing during winter dormancy of above‐ground tissues. Therefore, we reject the first part of Hypothesis 1, which implied root water uptake and below‐ground to above‐ground water transport to be strongly restricted during cold temperatures. Young‐Robertson et al. (2016) and Thalheimer et al. (2024) also detected root water uptake during winter for boreal deciduous trees and on apple trees, respectively. Nevertheless, the underlying mechanisms for root water uptake at the end of winter are still unclear. An active process from the vascular system seems possible, as roots appear to have no dormancy at soil temperatures above freezing (Malyshev et al. 2023). In addition, passive absorption through the bark of coarse roots is likely happening, and water may then move by diffusion following osmotic gradients (Cuneo et al. 2018). Our methodology does not allow the separation of active water uptake by fine roots and passive transport processes from the soil into coarse roots. However, our results demonstrate that the roots can absorb water during winter dormancy and supply other organs, such as the stem.

Surprisingly, ^2^H_2_O‐label was even detected in twigs and buds of *S. torminalis*, *F. sylvatica* and *P. sylvestris* at the end of the chilling phase. This finding illustrates that water allocation from stem to twigs and buds is happening for these species during winter at temperatures just above freezing. Water loss to the atmosphere and diffusion of water from neighbouring tissues to replenish the water loss could be a potential reason for the observed allocation of water from lower stem to twigs and buds under cold temperatures (Thalheimer et al. 2024). No ^2^H_2_O‐label, however, was found in the twigs and buds of *Q. petraea*, providing additional evidence that the winter water allocation processes of *Q. petraea,* a ring‐porous species, are fundamentally different from the other investigated species with different wood structures (Dai, Wang, and Wan 2020).

### 4.2 Deciduous Broad‐Leaf Species Differ in Water Allocation Strategies to Sustain Budburst

Temporal variations in tissue water content and ^2^H_2_O label uptake with increasing forcing conditions indicate different water allocation strategies from stem to twig and bud. Water flow from the stem's lower part into buds was already detected in *S. torminalis* during the chilling phase, and bud Δ^2^H was strongly associated with twigs Δ^2^H for all species but *F. sylvatica* during Experiment 1. This finding illustrates that at least some water flow is happening between twigs and buds before budburst, in line with what was observed by Walde et al. (2024). At the initiation of winter dormancy, water transport between tree tissues is restricted by low plasmodesmal and low passive water transport between cells due to high callose deposition and repression of aquaporin genes, respectively (Fouché et al. 2023; Rinne and Schoot 2003; Walde et al. 2024; Yooyongwech et al. 2008). Therefore, the water flow occurring between twigs and buds during the chilling phase could be driven by the degradation of callose and activation of aquaporins after a specific exposure to chilling conditions (Rinne, Kaikuranta, and Van der Schoot 2001; Rinne et al. 2011; Yooyongwech et al. 2008). Water allocation from distant tissues is involved in bud swelling and budburst of *S.* torminalis, even though more extensive water allocation processes from below‐ground to above‐ground organs are probably only initiated once the leaves are fully unfolded.

*F. sylvatica,* on the other hand, maintained bud water content and bud Δ^2^H throughout the forcing phase, which indicates that this species hydrates twigs from distant water sources, likely even from soil water during winter. However, water flow from twigs into buds of *F. sylvatica* during the forcing phase of this experiment was restricted, whereas the dormancy release mechanisms of the other species were probably already fulfilled (Fouché et al. 2023; Yooyongwech et al. 2009). This finding is consistent with previous work that showed the spring phenological development of *F. sylvatica* to be largely dependent on photoperiodic conditions (Basler and Körner 2012; Malyshev et al. 2018; Walde et al. 2022). The artificial photoperiod during the forcing conditions of this experiment (i.e., early March) was probably too short to trigger bud swelling as the photoperiod was just below 12 h during the forcing phase of Experiment 1 (see Fu et al. 2013; and Vitasse and Basler 2012).

We found a significant increase in twig and bud Δ^2^H of *Q. petraea* with increasing forcing, but twig and bud Δ^2^H were not significantly higher than the background. Thus, vascular transport processes appear to be restricted between stem and twig for *Q. petraea*, whereas the connection between twigs and buds seems unblocked. In contrast to *Q. petraea*, we traced some water flow between stem and twigs in the other species, which indicates species‐specific tree physiological characteristics during dormancy release, as predicted by the second part of Hypothesis 1. However, twig‐bud water flow in *Q. petraea* could also be provided in those small quantities through non‐vascular water transport, such as the apoplastic and symplastic pathways. Earlywood vessels of ring‐porous species are more prone to frost‐related embolisms than conifers and diffuse‐porous species (Dai, Wang, and Wan 2020; Sperry et al. 1994). Consequentially, ring‐porous species such as *Q. petraea* must rebuild earlywood vessels in spring to supply buds and twigs with water through the vascular system (Cochard and Tyree 1990; Essiamah and Eschrich 1986). The low Δ^2^H found in this species' bud and twig might be due to the overall hydraulic capacity limitations in early spring. However, we cannot rule out additional non‐vascular water transport system restrictions.

### 4.3 Atmospheric Water Uptake Into Above‐Ground Tissues Before Bud Break Is Possible

Following Hypothesis 2, *Q. petraea*, *F. sylvatica* and *P. sylvestris* showed substantial incorporation of atmospheric ^2^H_2_O‐label into stem, twig and bud tissue before budburst. This finding indicates that trees can absorb water through the surface of above‐ground organs, as previously found for *P. abies* during late winter (Mayr et al. 2014). If the water transport from root to restricted, atmospheric water uptake could be a hydration pathway at air conditions close to water vapour saturation (e.g., early morning during spring).

The absorption of water into twigs and stems could have occurred through lenticels, which were shown to facilitate air moisture uptake into the bark (Beckett et al. 2024; Groh, Hübner, and Lendzian 2002; Rosner and Morris 2022). However, our study did not differentiate between the different tissues of the buds. Therefore, additional research is needed to prove whether buds incorporate labelled water into their bud scales only or if this water can also be allocated in leaf primordia and meristem cells.

After a short exposition to forcing conditions, we found significant differences in Δ^2^H among tissues between *Q. petraea* and *F. sylvatica* (i.e., deciduous broad‐leaf) and the evergreen coniferous species (i.e., *P. sylvestris*) with a higher ^2^H_2_O‐label uptake into buds than the stem for both deciduous broad‐leaf species. This difference in ^2^H_2_O‐label uptake is partially mirrored by the quantified amount of incorporated label, where we found substantial differences between stem and acropetal tissues for *Q. petraea* but not for *F. sylvatica*. Interestingly, *F. sylvatica* showed a higher incorporation of label into the stem and a lower incorporation of label into twigs and buds than *Q. petraea*, which suggests that the water flow between stem and acropetal tissues is more severely imposed by internal restrictions in *Q. petraea* due to immature xylem in early spring (Essiamah and Eschrich 1986). The stem tissues of *Q. petraea* stems seem well supplied with water from the roots during this time, while the acropetal tissues of this species cannot fully compensate for the water lost by transpiration. In contrast, root–stem conduits of *F. sylvatica* could be less developed during this time. Another reason for the higher incorporation of isotopic label into *F. sylvatica* sapling stems could be the differences in water conductivity of the bark of the two species. *P. sylvestris* showed a tight relationship between twig water content and Δ^2^H in Experiment 1 (i.e., below‐ground ^2^H_2_O‐labelling) and no significant differences between bud and stem in Experiment 2 (atmospheric ^2^H_2_O‐labelling), which indicates that the vascular system of this species is fully operational at least few days after exposure to forcing temperatures. Further evidence for a functional vascular system is provided by the low amount of incorporated isotopic label found in *P. sylvestris* tissues compared to the tissues of both deciduous broad‐leaf species. Thus, the presence of needles for *P. sylvestris* might have facilitated the release of incorporated water vapour from plant tissues to the atmosphere after the above‐ground labelling of Experiment 2 before the plant tissues were sampled. In contrast to Hypothesis 3, we did not find the deciduous broad‐leaf species *F. sylvatica* and *Q. petraea* to take up carbon by bark photosynthesis in traceable quantities before budburst.

We found significantly higher *δ* ^13^C values in all investigated tissues for the evergreen conifer species *P. sylvestris* after the ^13^CO_2‐_labelling of Experiment 2. This finding reveals further evidence that evergreen conifer species such *P. sylvestris* begin autotrophic photosynthesis by the last years' needles and starts reallocating carbon‐containing assimilates fixed of the canopy into buds and stems shortly after exposure to forcing conditions. Nevertheless, bark photosynthesis could have also contributed to the ^13^C excess of the stem of *P. sylvestris* in this experiment (reviewed in Ávila, Herrera, and Tezara 2014). No correlation between bud water content and bud Δ^2^H was found in Experiment 1. Therefore, the investigated evergreen conifer, *P. sylvestris,* likely initiated stem‐to‐twig water transport to supply photosynthesis shortly after exposure to forcing conditions, thereby increasing twig water content. Even though water flow from twigs into buds was likely occurring at that time, bud swelling was not observed for this species for at least 3 weeks after exposure to forcing conditions.

## 5 Conclusion and Perspective

Overall, the study provides new insights into water uptake and plant internal water allocation processes of structurally different Central European tree species during winter. We illustrate that root water uptake and allocation to above‐ground tissue happens at temperatures just above freezing thereby declining the first part of Hypothesis 1, likely to maintain tissue hydration before budburst. Nevertheless, there were, in agreement with the second part of Hypothesis 1, substantial differences in water allocation among the studied tree species at the initiation of spring, which we attributed to differences in wood anatomical traits, dormancy release drivers and the presence or absence of foliage. Further, in accordance with our second hypothesis, atmospheric water may contribute to tissue hydration during winter dormancy and early spring. However, contrary to our third hypothesis, the investigated deciduous tree species did not take up carbon in traceable quantities before budburst. Our study demonstrates that tree water uptake and water allocation during winter are more substantial and complex than previously thought.

## Conflicts of Interest

The authors declare no conflicts of interest.

## Supporting information

Supporting information.

## Data Availability

The data that support the findings of this study are available from the corresponding author upon reasonable request.
