# Supplementary material for: Stable Isotope Labelling Reveals Water and Carbon Fluxes in Temperate Tree Saplings Before Budbreak
Source: Plant Cell Environ. 2024 Oct 1;48(1):805–17. doi: 10.1111/pce.15173 (PMC11615418; doi:10.1111/pce.15173)
Supplement: Supplementary file 1 — Supporting information. [file PCE-48-805-s001.docx]

Supplementary Information:
Before buds break – Stable isotope labeling reveals water and carbon allocation dynamics in temperate tree saplings

# Supplementary calculations

## Calculation of relative air humidity

Relative air humidity was measured inside the climate chamber but not inside the tent. Therefore, we calculated the saturation vapor pressure inside the tent (e_s_) using the August-Roche-Magnus formula (equation 1), where T is the temperature inside the climate chamber in °C.

| $e_{s}=6.11\cdot exp\left( \frac{17.625\cdot T}{T+243.04} \right)$ | (S1) |
| --- | --- |

Vapor pressure (e) was calculated as the partial pressure of water vapor in the air (p_H2O_) using equation 2, where x_H2O_ is the fraction of H_2_O in the atmosphere in ppm (measured by L2120-i, Picarro Inc., Santa Clara, CA, USA), and p is the average atmospheric pressure on April 5, 2022 measured at the meteorological station of the institute (~95.0 kPa).

| $e=p_{H2O}=x_{H2O}\cdot p$ | (S2) |
| --- | --- |

Then, relative air humidity (RH) inside the tent was calculated from e and e_s_ using equation 3.

| $RH=\frac{e}{e_{s}}\cdot100$ | (S3) |
| --- | --- |

## Calculation of incorporated label fraction into plant tissues

For a better comprehension of the ^2^H_2_O-label excess, the mean fraction of the label incorporated into plant tissue (µ_incorp_) was approximated for experiment 1 from the mean isotopic composition of the soil $\left( \mu_{\delta^{2}H_{soil}} \right),$ directly determined after the below-ground labeling, background $(\mu_{\delta^{2}H_{background}}$), and sample $(\mu_{\delta^{2}H_{sample}}$, equation S4).

| $\mu_{incorp}(\%)=\frac{\mu_{\delta^{2}H_{sample}}-\mu_{\delta^{2}H_{background}}}{\mu_{\delta^{2}H_{soil}}-\mu_{\delta^{2}H_{background}}}=\frac{\mu_{\Delta^{2}H}}{\mu_{\delta^{2}H_{soil}}-\mu_{\delta^{2}H_{background}}}$ | (S4) |
| --- | --- |

The standard deviation of the label incorporated into plant tissue (σ_incorp_) was calculated as a propagation of uncertainty of mean and standard deviation of the isotopic composition of the soil, background, and sample (equation S5).

| $\sigma_{incorp}\left( \% \right)=\sqrt{\left( \frac{{\partial\mu}_{incorp}}{{\partial\mu}_{\Delta^{2}H}} \right)^{2}\cdot{\sigma_{\Delta^{2}H}}^{2}\cdot\left( \frac{{\partial\mu}_{incorp}}{{\partial\mu}_{\delta^{2}H_{background}}} \right)^{2}{\cdot\sigma_{\delta^{2}H_{background}}}^{2}\cdot\left( \frac{{\partial\mu}_{incorp}}{{\partial\mu}_{\delta^{2}H_{soil}}} \right)^{2}\cdot{\sigma_{\delta^{2}H_{soil}}}^{2}}$ | (S5) |
| --- | --- |

The calculation of the three partial derivatives led to equation S6.

| $\sigma_{incorp}(\%)=\sqrt{\begin{aligned} \left( \frac{1}{\mu_{\delta^{2}H_{soil}}-\mu_{\delta^{2}H_{background}}} \right)^{2}{\cdot{\sigma_{\Delta^{2}H}}^{2}\cdot\left( \frac{\mu_{\Delta^{2}H}}{\left( \mu_{\delta^{2}H_{soil}}-\mu_{\delta^{2}H_{background}} \right)^{2}} \right)}^{2}\cdot{\sigma_{\delta^{2}H_{background}}}^{2}\cdot\\ \left( \frac{-\mu_{\Delta^{2}H}}{\left( \mu_{\delta^{2}H_{soil}}-\mu_{\delta^{2}H_{background}} \right)^{2}} \right)^{2}\cdot{\sigma_{\delta^{2}H_{soil}}}^{2} \end{aligned}}$ | (S6) |
| --- | --- |

For experiment 2, the mean fraction of the label incorporated into plant tissue and the corresponding standard deviation was calculated similarly to experiment 1.

# Supplementary tables

**Table S1:** Characterization of the potted tree saplings. Height±SD corresponds to the height and standard deviation of all saplings investigated in the experiment of the respective species.

|  | **Height±SD** | **Age** | **Seed origin** | **Pot size** | **Wood anatomy** |
| --- | --- | --- | --- | --- | --- |
| *F. sylvatica* | 50±9 cm | 5 years | 47°11’ N, 7°32’ E | 4 l | Diffuse-porous |
| *P. sylvestris* | 87±9 cm | 5 years | 46°18’ N, 7°37’ E | 3 l | Tracheid |
| *Q. petraea* | 83±16 cm | 5 years | 47°26’ N, 7°47’ E | 3 l | Ring-porous |
| *S. torminalis* | 83±14 cm | 2 years | 47°44’ N, 8°34’ E | 4 l | Diffuse-porous |

**Table S2:** Approximation of the mean label fraction (%) incorporated into the plant tissue and corresponding standard deviation (µ±σ) at the initiation of the forcing phase of experiment 1. The label fraction was calculated from mean and corresponding standard deviation of the isotopic composition (δ^2^H-values in ‰) of label excess, labeled soil, and background using equations S4 and S6.

| **Species** | **Tissue** | **Incorporated label (µ±σ)** | **Label excess (µ±σ)** | **Labeled soil (µ±σ)** | **Background (µ±σ)** |
| --- | --- | --- | --- | --- | --- |
|  |  | fraction (%) | Δ^2^H (‰) | δ^2^H_soil_ (‰) | δ^2^H_background_ (‰) |
| *S. torminalis* | Buds | 2.2±1.1 | 10±2 | 399±212 | -38±2 |
|  | Twigs | 1.7±0.8 | 7±2 | 399±212 | -38±4 |
|  | Stem | 14.2±6.2 | 69±12 | 399±212 | -86±2 |
| *Q. petraea* | Buds | -0.1±0.1 | 0±4 | 273±213 | -29±5 |
|  | Twigs | -1.4±1.0 | -4±4 | 273±213 | -26±5 |
|  | Stem | 19.3±11.4 | 70±4 | 273±213 | -88±2 |
| *F. sylvatica* | Buds | 2.6±0.5 | 12±2 | 423±52 | -39±4 |
|  | Twigs | 2.7±0.3 | 12±5 | 423±52 | -40±6 |
|  | Stem | 11.1±1.2 | 55±10 | 423±52 | -71±4 |
| *P. sylvestris* | Buds | 1.6±0.5 | 10±6 | 554±208 | -63±2 |
|  | Twigs | 2±0.7 | 12±12 | 554±208 | -45±6 |
|  | Stem | 10.5±3.4 | 67±11 | 554±208 | -83±3 |

**Table S3:** Approximation of the mean label fraction (%) incorporated into the plant tissue and corresponding standard deviation (µ±σ) of experiment 2. The label fraction was calculated from mean and corresponding standard deviation of the isotopic composition (δ^2^H-values in ‰) of label excess, labeled water vapor, and background using equations S4 and S6.

| **Species** | **Tissue** | **Incorporated label (µ±σ)** | **Label excess (µ±σ)** | **Labeled vapor (µ±σ)** | **Background (µ±σ)** |
| --- | --- | --- | --- | --- | --- |
|  |  | fraction (%) | Δ^2^H (‰) | δ^2^H_vapor_ (‰) | δ^2^H_background_ (‰) |
| *Q. petraea* | Buds | 12.4±6 | 64±32 | 491±247 | -22±5 |
|  | Twigs | 10.5±5.1 | 53±21 | 491±247 | -17±5 |
|  | Stem | 3.7±1.6 | 21±8 | 491±247 | -78±2 |
| *F. sylvatica* | Buds | 9.8±5.0 | 51±9 | 491±247 | -26±4 |
|  | Twigs | 7.2±3.4 | 38±7 | 491±247 | -31±6 |
|  | Stem | 7.2±3.3 | 40±45 | 491±247 | -56±4 |
| *P. sylvestris* | Buds | 3.7±1.7 | 20±5 | 491±247 | -50±2 |
|  | Twigs | 3.7±1.7 | 20±18 | 491±247 | -36±6 |
|  | Stem | 2.3±1.0 | 13±8 | 491±247 | -67±3 |

# Supplementary figures


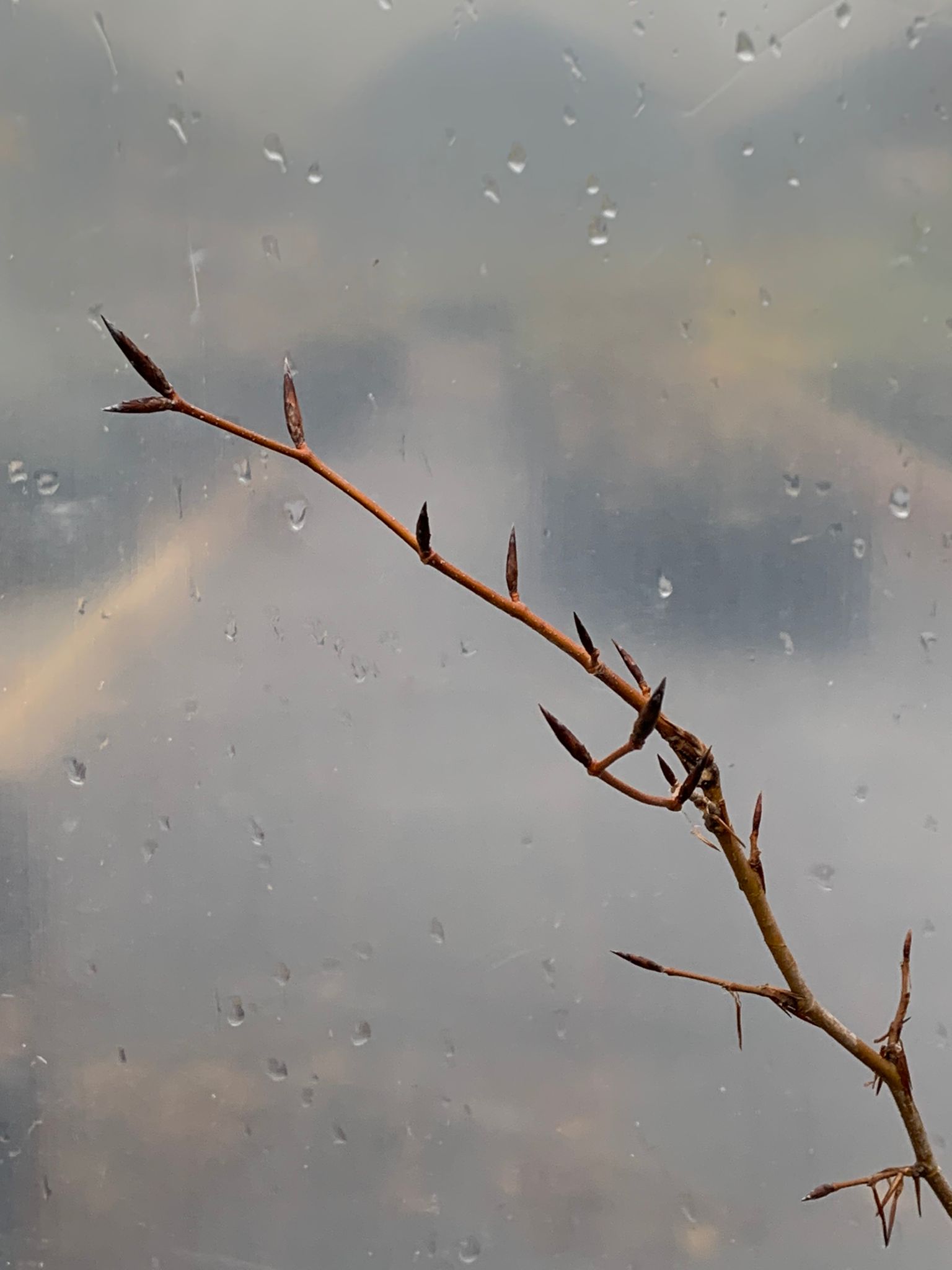

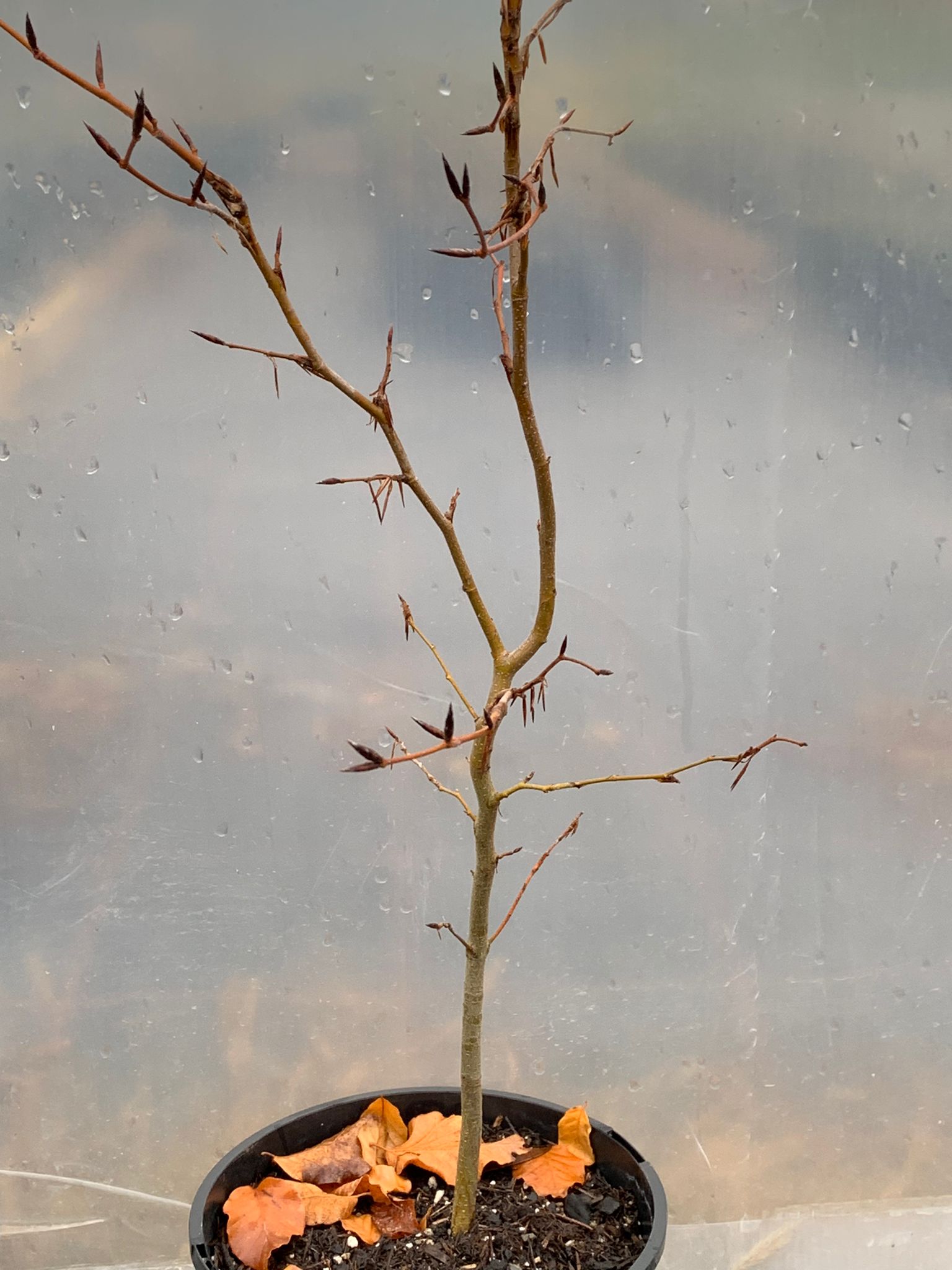

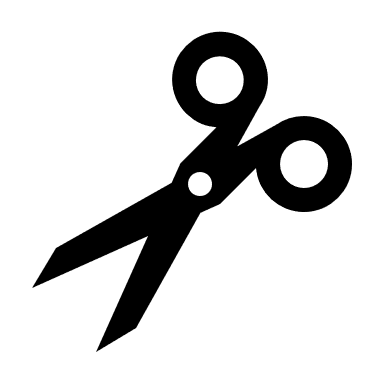

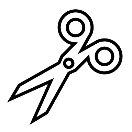

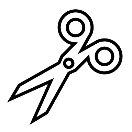

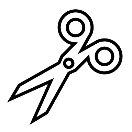


**Figure S1:** Illustration of the stem sampling (left) and twig and bud sampling (right). 2 cm of stem samples were collected at about 3-5 cm above ground, while at least 5 twigs and buds were sampled from the uppermost parts of the sapling. Buds were abscised at about 1-2 mm above the twig-bud interface (black scissors). Twigs were abscised 1-2 cm below the bud (white scissors).


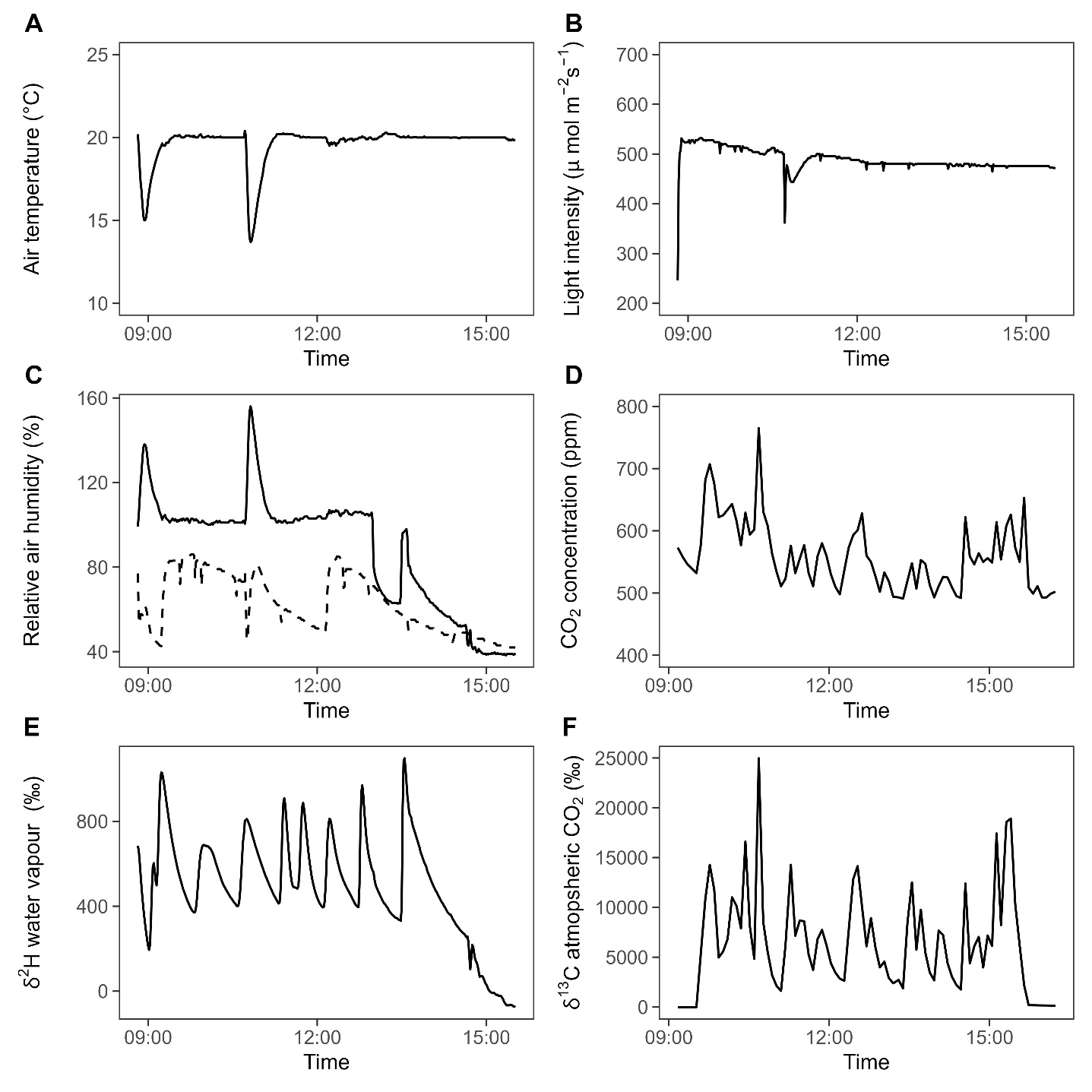


**Figure S2:** Illustration of the experimental conditions of experiment 2 during the atmospheric labeling. (A) Air temperature and (B) light intensity were measured inside the climate chamber. (C) Relative air humidity (RH) is represented as dashed line for the measurement inside the chamber, whereas the solid lines represent the estimated RH inside the tent placed inside the climate chamber. (D) CO_2_ concentration and (E, F) stable isotopes were measured inside the tent placed. RH in the tent was calculated from saturation vapor pressure and water vapor pressure at the air temperature of the climate chamber.


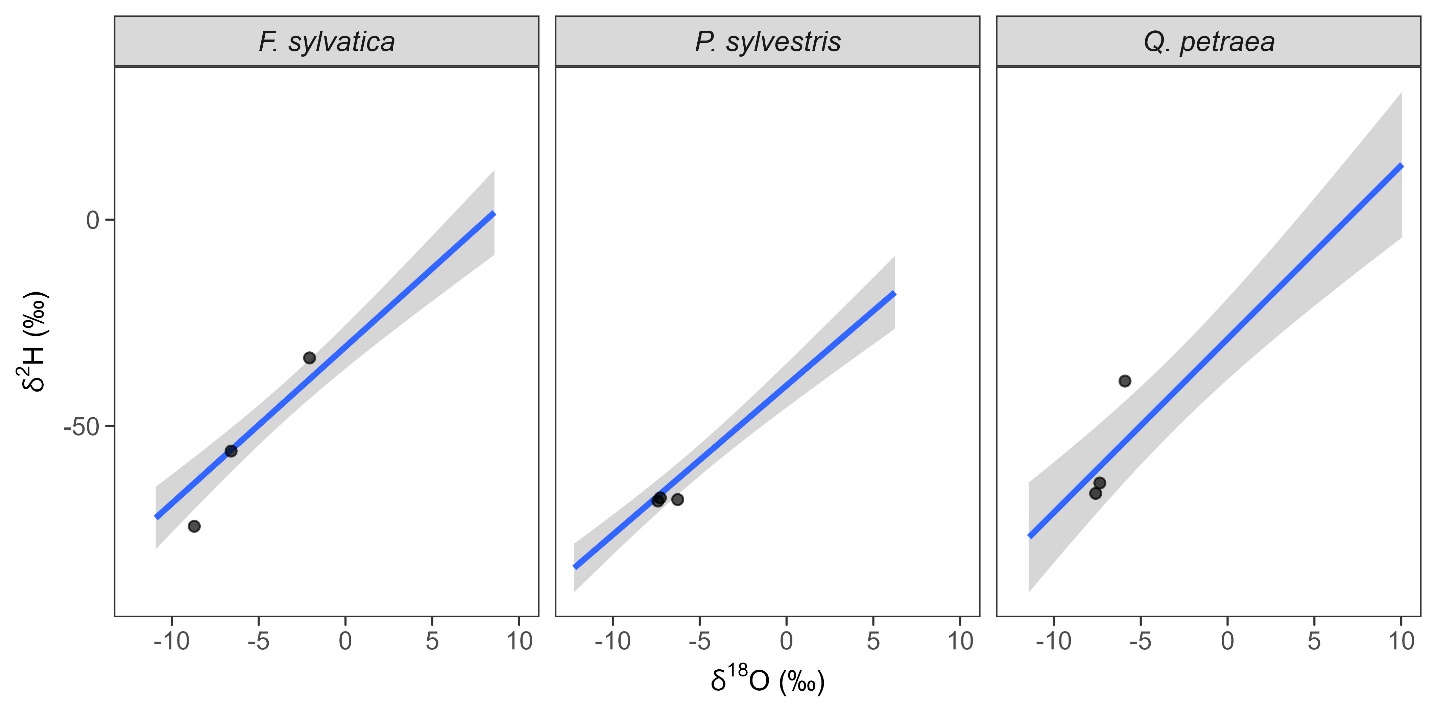


**Figure S3:** Illustration of the soil water's isotopic composition in experiment 2 after the atmospheric labeling with water vapor enriched in ^2^H_2_O displayed as black dots. Regression lines with corresponding 0.95 confidence intervals depict the linear relationship between δ^2^H and δ^18^O determined from tissue water of three non-labeled saplings after seven days of the chilling phase and from three saplings at the last sampling date of the forcing phase.

**
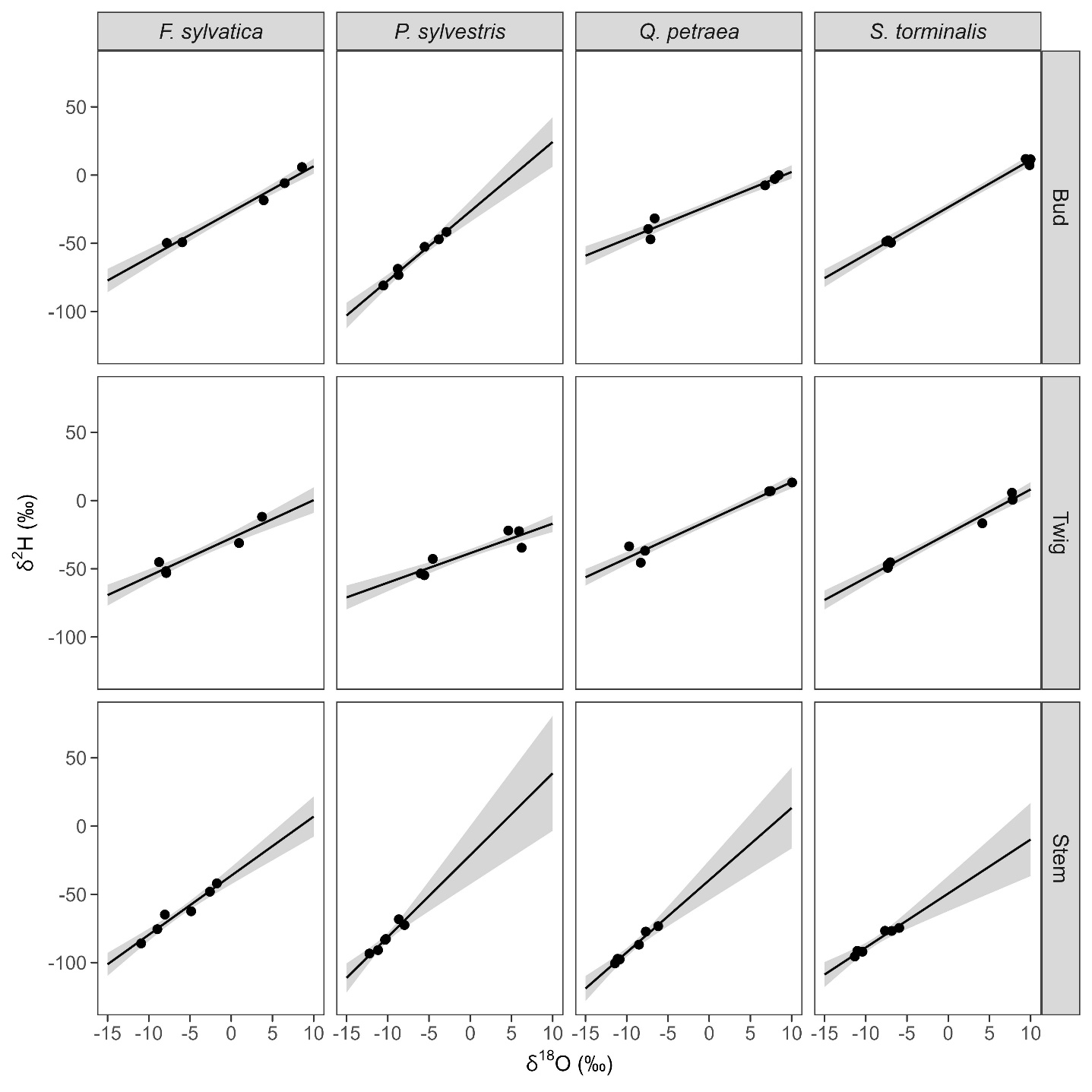
**

**Figure S4:** Illustration of the species- and tissue-specific linear relationship with corresponding 0.95 confidence intervals between δ^2^H and δ^18^O. The linear relationship was determined from three non-labeled saplings after seven days of the chilling phase and from three saplings at the last sampling date of the forcing phase of experiment 1 (black dots).


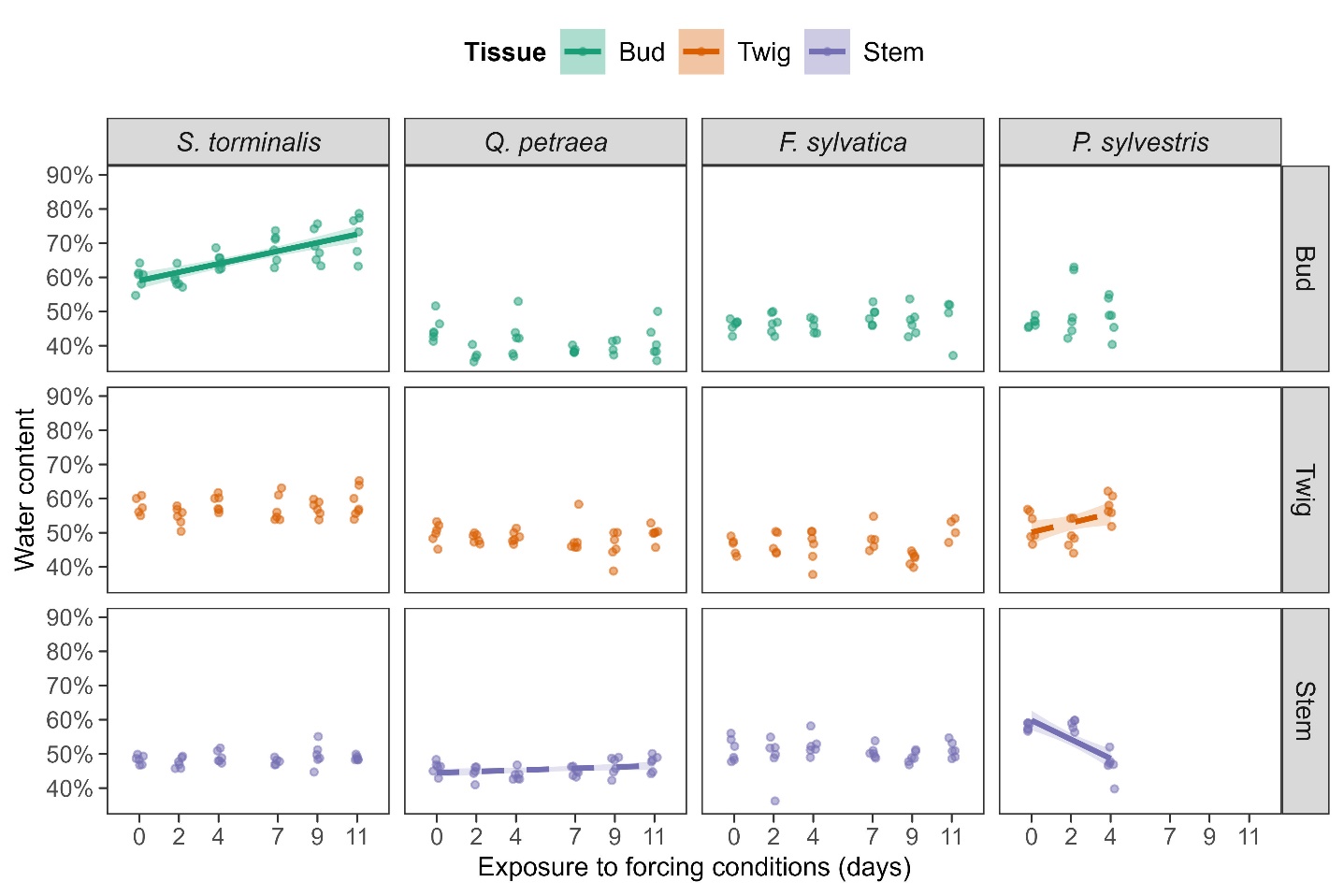


**Figure S5:** Change in water content with increasing forcing conditions in experiment 1 for each study species and tissue. Regression lines with corresponding 0.95 confidence intervals depict the direction of the correlations. Solid lines represent significant correlations (*P*≤0.050), while dashed lines represent correlations with weak, yet not significant, evidence (0.050<*P*<0.100).


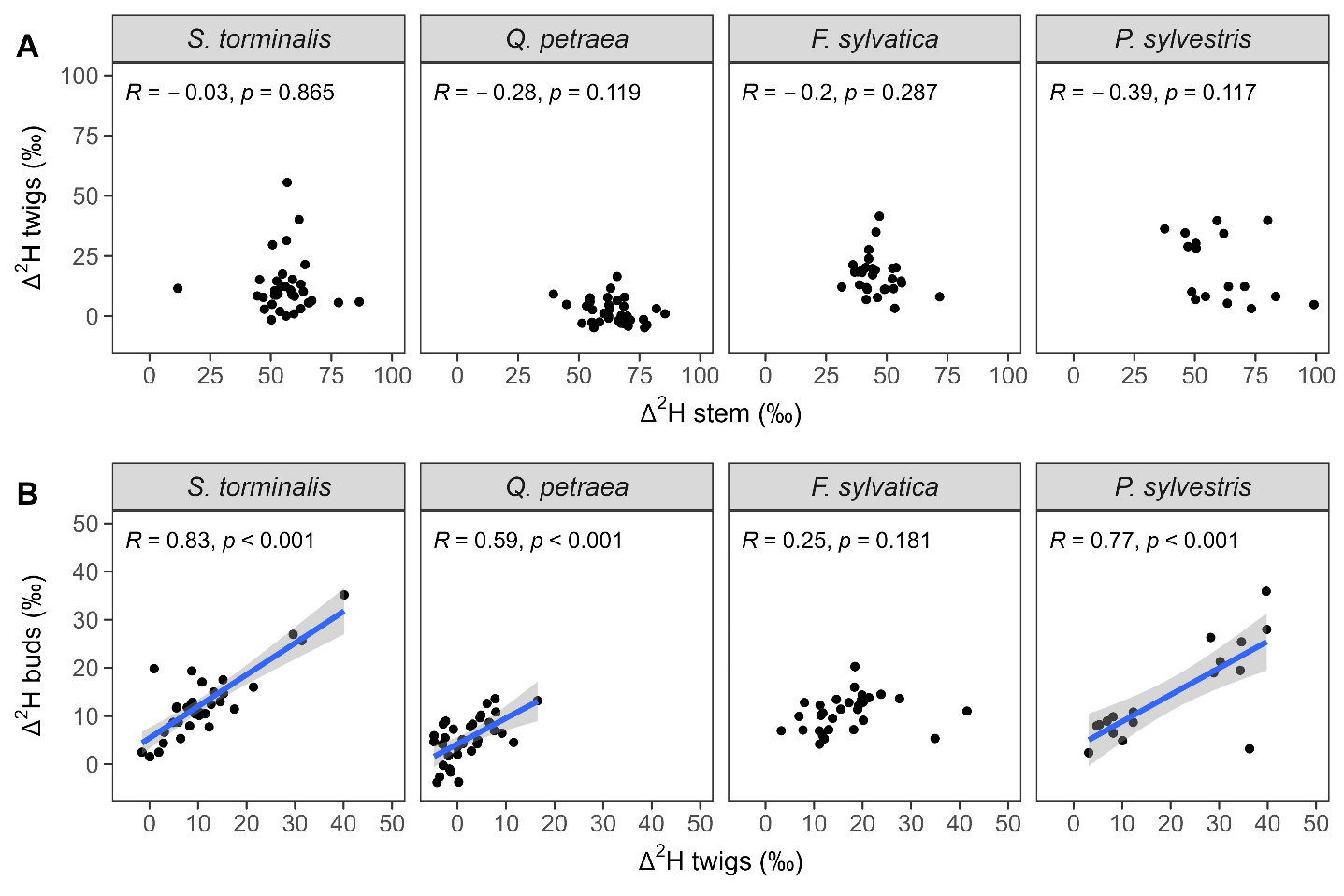


**Figure S6:** (A) Correlation of stem Δ^2^H with twig Δ^2^H and (B) correlation of twig Δ^2^H with bud Δ^2^H in experiment 1 for each study species. Regression lines with corresponding 0.95 confidence intervals depict represent significant correlations (*P*≤0.050).
